# ACE2, TMPRSS2, and furin gene expression in the airways of people with asthma—implications for COVID-19

**DOI:** 10.1016/j.jaci.2020.05.013

**Published:** 2020-07

**Authors:** Peter Bradding, Matthew Richardson, Timothy S.C. Hinks, Peter H. Howarth, David F. Choy, Joseph R. Arron, Sally E. Wenzel, Salman Siddiqui

**Affiliations:** a National Institute for Health Research (NIHR) Leicester Biomedical Research Centre (Respiratory theme) and College of Life Sciences, University of Leicester, Leicester, United Kingdom; b Respiratory Medicine Unit and National Institute for Health Research (NIHR) Oxford Biomedical Research Centre (BRC), Nuffield Department of Medicine Experimental Medicine, University of Oxford, Oxford, United Kingdom; c Clinical and Experimental Science, Faculty of Medicine, University of Southampton and National Institute for Health Research (NIHR) Southampton Biomedical Research Centre, University Hospital Southampton NHS Foundation Trust, Southampton, United Kingdom; d Genentech, South San Francisco, Calif; e University of Pittsburgh Asthma Institute at UPMC/UPSOM, Pittsburgh, Pa

To the Editor:

Coronavirus disease 2019 (COVID-19) is caused by a novel zoonotic coronavirus known as severe acute respiratory syndrome coronavirus 2 (SARS-CoV-2) and has been identified as a pandemic by the World Health Organization. Several risk factors have been identified for severe COVID-19–associated pneumonia including increased age and the presence of comorbidities, in particular diabetes, cardiovascular disease, and tobacco smoking.^1^ However, a number of reports have failed to identify excess risk in patients with respiratory airway diseases such as asthma.^2^

SARS-CoV-2 infects people by binding to the angiotensin-converting enzyme 2 (ACE2) receptor, a transmembrane endopeptidase that cleaves both angiotensin 1 and 2, and which is expressed by epithelial cells in several organs including the airways. Cofactors facilitating SARS-CoV-2 infectivity are the transmembrane peptidase serine 2 (TMPRSS2), which cleaves the SARS-CoV-2 spike protein, and possibly the protease furin.^3^ Understanding the expression of ACE2, TMPRSS2, and furin in the airways of people with asthma may help determine whether asthma itself or treatment with inhaled or oral corticosteroids may alter susceptibility to SARS-CoV-2 infection and potentially related disease severity. We have therefore explored the RNA expression of ACE2, TMPRSS2, and furin in human bronchial brushes and biopsies from previously described cohorts of people with asthma of varying corticosteroid treatment intensity (as an index of severity) and healthy controls.

Airway brushes and biopsies were collected at bronchoscopy with written informed consent and ethical approvals. Airway brushes were placed into RNAprotect and airway biopsies from the second- to fifth-generation airways were placed into RNAlater. Bronchial brush ACE2 expression data were available from 356 patients (88 healthy volunteers and 268 patients with asthma [mild to moderate asthma, 125; severe asthma, 143]), across 5 asthma/healthy volunteer cohorts, Leicester, UK (n = 34),^4^ the multicenter Bronchoscopic Exploratory Research Study of Biomarkers in Corticosteroid-refractory Asthma (n = 54),^5^ the Severe Asthma Research Program cohort (n = 154),^6^ and Southampton, UK (n = 114).^7^^,^^8^ Bronchial biopsy ACE2 expression data (n = 94) were available in 17 healthy volunteers and 77 patients with asthma from the Leicester and Bronchoscopic Exploratory Research Study of Biomarkers in Corticosteroid-refractory Asthma cohorts. For bronchial brush data, the 5 data sets were combined into a single data set after first adjusting for batch effects by separately mean centering each of the 5 data sets. The same method was applied to the biopsy data. The Wilcoxon rank-sum test and the Kruskal-Wallis test were used to test for between-group differences in ACE2 expression. The Spearman rank correlation was used when reporting correlations. All analyses were performed using R statistics, version 3.6.0, and figures were generated in GraphPad Prism 8.1.2 (GraphPad Software, San Diego, Calif).

**Fig 1:**
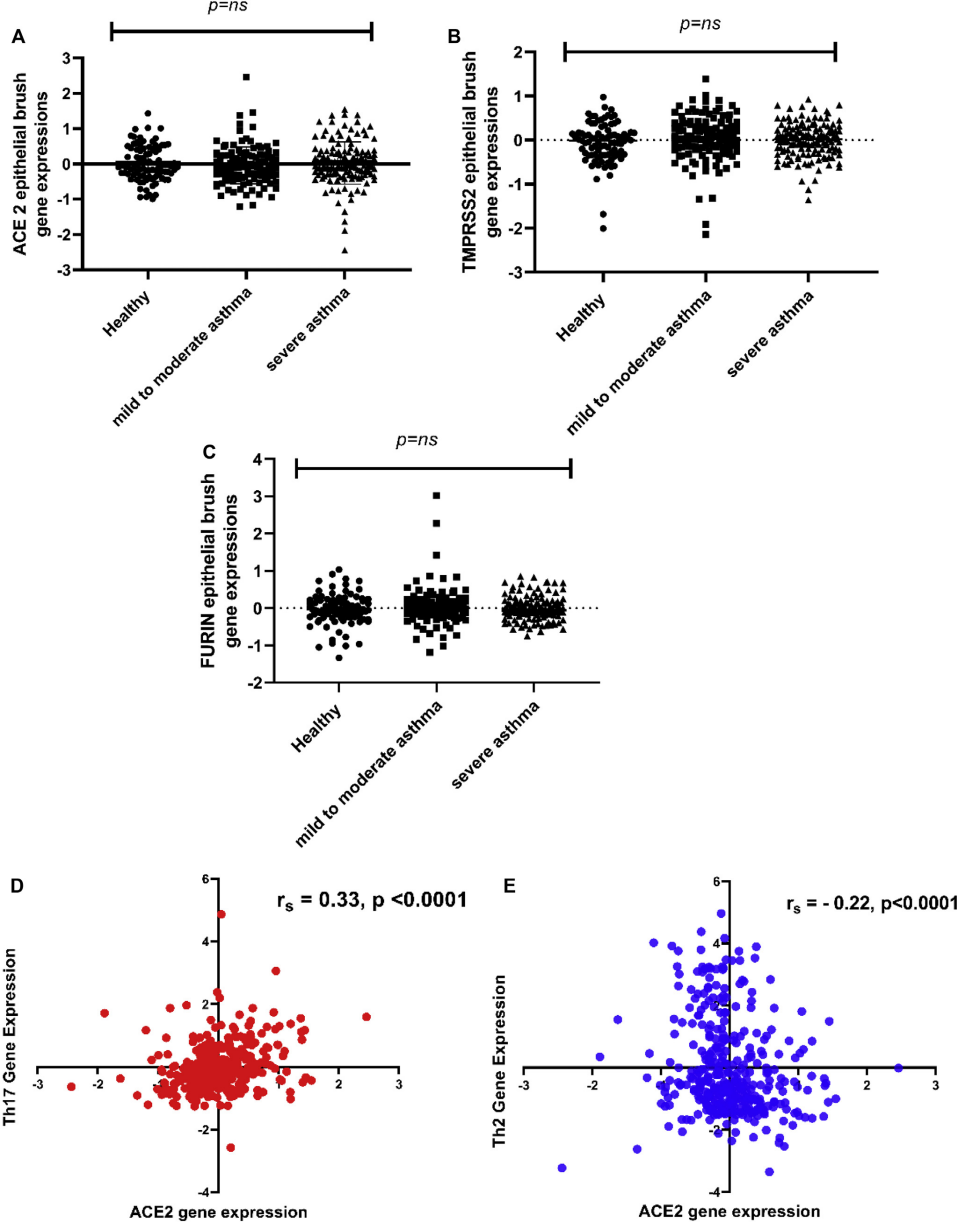
**A-C**, ACE2, TMPRSS2, and furin gene expression in bronchial brush samples is not increased in patients with mild to moderate or severe asthma compared with healthy controls. **D**, There is a weak positive correlation between ACE2 gene expression and a T_H_17–dependent gene expression signature in bronchial brushes samples (healthy control and patients with asthma combined). **E**, There is a weak inverse correlation between ACE2 gene expression and a T_H_2-dependent gene expression signature in bronchial brushes samples (healthy control and patients with asthma combined).

The clinical characteristics of the participants (median [Q1:3]) for healthy volunteers versus people with asthma were respectively as follows: age—25 (22-34) versus 40 (27-49) years, FEV_1_% predicted—101 (93-109) versus 71.5 (58-88), and FEV_1_/forced vital capacity—82 (78-85) versus 72 (62-79). Overall, there was no difference for ACE2, TMPRSS2, or furin mRNA expression between people with asthma compared with healthy controls (*P* = .96), no significant differences in ACE2 expression between males and females, and no correlation between ACE2 gene expression and age (data not shown). There were no differences in ACE2, TMPRSS2, or furin gene expression between healthy volunteers and people with mild to moderate and severe asthma (Fig 1, *A-C*). ACE2, TMPRSS2, or furin gene expressions were not correlated.

There were weak but highly significant inverse and positive correlations between ACE2 expression and the expression of T_H_2-dependent and IL-17 (T_H_17)-dependent epithelial gene signatures, respectively, defined as previously^4^ (Fig 1, *D* and *E*). Similar observations were noted in bronchial biopsies, with no differences in ACE2 gene expression between healthy volunteers and patients with mild to moderate asthma and patients with severe asthma (*P* = .43) (not shown).

These data would suggest that differences in ACE2, TMPRSS2, and furin epithelial and airway gene expression are unlikely to confer enhanced COVID-19 pneumonia risk in patients with asthma across all treatment intensities and severity. It is therefore possible that the risk of severe COVID-19 pneumonia is no greater than the background population risk in patients with asthma in the absence of other known risk factors such as diabetes and cardiovascular disease. This would support current guidance on the use of inhaled steroids and rescue prednisolone in patients with asthma who experience exacerbations during the COVID-19 pandemic.

A previous mouse model of infection demonstrated that ACE2 inhibits neutrophil infiltration and lung inflammation by limiting IL-17 signaling by reducing the activity of the signal transducer and activator of transcription 3 pathway.^9^ However, our observations in bronchial brush airway epithelial cells identified a positive correlation between ACE2 gene expression and a previously described IL-17–dependent gene expression signature, with an inverse association with T_H_2 gene expression. It is possible that ACE2 protein expression in the airways might not mirror the RNA expression, which is a limitation of our study. Furthermore, the precise relationship between other host immunoregulatory factors that may modify the risk of severe COVID-19 pneumonia and asthma, as well as corticosteroid exposure, which may induce T_H_17 immunity in asthma, have not been examined here directly. However, our data are in keeping with a recent report demonstrating that in nasal brushings from children, ACE2 expression was inversely correlated with markers of type 2 immunity, with no influence of sex or use of nasal corticosteroids.^10^ In the same article, it was shown that segmental bronchial allergen challenge in adults with mild asthma led to decreases in ACE2 expression, and that IL-13 reduces ACE2 expression on cultured bronchial epithelial cells.

In summary, these data suggest that it will be important to understand further the effects of T_H_2 and IL-17–driven inflammation, and of inhaled corticosteroids on airway epithelial cell ACE2 expression, and the susceptibility of these cells to infection and replication by SARS-CoV-2.
